# Evaluation of Nephroprotective and Immunomodulatory Activities of Antioxidants in Combination with Cisplatin against Murine Visceral Leishmaniasis

**DOI:** 10.1371/journal.pntd.0001629

**Published:** 2012-05-01

**Authors:** Meenakshi Sharma, Rakesh Sehgal, Sukhbir Kaur

**Affiliations:** 1 Department of Zoology, Panjab University, Chandigarh, India; 2 Department of Parasitology, Postgraduate Institute of Medical Education and Research, Chandigarh, India; McGill University, Canada

## Abstract

**Background:**

Most available drugs against visceral leishmaniasis are toxic, and growing limitations in available chemotherapeutic strategies due to emerging resistant strains and lack of an effective vaccine against visceral leishmaniasis deepens the crisis. Antineoplastic drugs like miltefosine have in the past been effective against the parasitic infections. An antineoplastic drug, cisplatin (cis-diamminedichloroplatinum II; CDDP), is recognized as a DNA-damaging drug which also induces alteration of cell-cycle in both promastigotes and amastigotes leading to cell death. First *in vivo* reports from our laboratory revealed the leishmanicidal potential of cisplatin. However, high doses of cisplatin produce impairment of kidney, which can be reduced by the administration of antioxidants.

**Methodology/Principal Findings:**

The present study was designed to evaluate the antileishmanial effect of cisplatin at higher doses (5 mg and 2.5 mg/kg body weight) and its combination with different antioxidants (vitamin C, vitamin E and silibinin) so as to eliminate the parasite completely and reduce the toxicity. In addition, various immunological, hematological and biochemical changes induced by it in uninfected and *Leishmania donovani* infected BALB/c mice were investigated.

**Conclusion/Significance:**

A significant reduction in parasite load, higher IgG2a and lower IgG1 levels, enhanced DTH responses, and greater concentration of Th1 cytokines (IFN-γ, IL-2) with a concomitant down regulation of IL-10 and IL-4 pointed towards the generation of the protective Th1 type of immune response. A combination of cisplatin with antioxidants resulted in successful reduction of nephrotoxicity by normalizing the enzymatic levels of various liver and kidney function tests. Reduction in parasite load, increase in Th1 type of immune responses, and normalization of various biochemical parameters occurred in animals treated with cisplatin in combination with various antioxidants as compared to those treated with the drug only. The above results are promising as antioxidants reduced the potential toxicity of high doses of cisplatin, making the combination a potential anti-leishmanial therapy, especially in resistant cases.

## Author Summary

Leishmaniasis, a neglected tropical disease (NTD) caused by *Leishmania*, has been put on the World Health Organization agenda for eradication as a part of their Special Programme for Tropical Diseases Research. Visceral leishmaniasis (VL) is a life-threatening disease when no treatment is given. Most of the drugs still used to treat VL are often expensive, difficult to administer, have serious side effects, and several are becoming ineffective because of increasing parasite resistance. Cisplatin is a first-generation platinum-containing drug, used in the treatment of various solid tumors. We have for the first time characterized the *in vivo* effect of cisplatin in murine experimental visceral leishmaniasis, but at higher doses it is nephrotoxic. Considering the above findings, the present study was designed to evaluate the protective efficacy of the drug in combination with various antioxidants to reduce or prevent cisplatin-induced nephrotoxicity. Drug treatment induces a higher secretion of Th1 cytokines, diminution in parasite burden, and the supplementation of antioxidants which are antagonists of the toxicity helps in reducing the nephrotoxicity.

## Introduction

Pentavalent antimonial compounds like sodium stibogluconate and N-methylglucamine antimoniate have been the mainstay of antileishmanial therapy [1]. They remain the conventional treatment of children and adults all over the world except in Bihar (India) where Sb is no longer useful owing to high failure rates due to resistance [2], [3] and also have the disadvantage of toxicity, parenteral administration and need for long duration of therapy [4]. Secondary treatment regimens with amphotericin B and pentamidine are effective but these are also parenteral, have to be administered for prolonged periods and therefore, are expensive and potentially toxic [2]. Liposomal formulations of amphotericin B target the cells that host the parasite and have decreased nephrotoxicity but are prohibitively costly. Paromomycin have advantages of high level of efficacy and low rates of adverse reaction, but the drawback is its high cost [5]. Oral drugs sitamaquine (WR 6020) and miltefosine are the two promising oral antileishmanial compounds. Miltefosine (hexadecylphosphocholine) is a membrane activating alkyl phospholipid, having cure rates of approximately 90–95%. It has an obvious advantage in being an active oral agent and hospitalization is thus not required [3] but is teratogenic in animals [3] so cannot be used in pregnant women. Considering the fact that therapeutic interventions against visceral leishmaniasis (VL) are limited and facing serious concerns of toxicity, high cost and emerging resistance, there is a greater interest in new drug developments which are cost effective, efficient and easily available to people suffering from leishmaniasis.

An antineoplastic drug, cisplatin (cis-diamminedichloroplatinum II; CDDP) a platinum-containing compound, is recognized as a DNA-damaging drug [6] and is known to augment the cytotoxic T-lymphocyte mediated antitumor immunity [7], [8]. It has been found to have antileishmanial activity *in vitro* at a concentration of 0.25–64 µM and has been shown to lead towards an apoptosis like cell death of both promastigotes and amastigotes [9]. First *in vivo* report from our laboratory also showed a significant reduction in parasite load and enhanced DTH responses which suggested the generation of the cell-mediated immune responses. Though the protective efficacy of the drug [10] was demonstrated, it could not completely eliminate the parasite at low dosages of 0.5 and 1 mg/kg b.wt.

In spite of its good antineoplastic activity against various cancer treatments, its clinical use was rapidly limited due to unexpected and very severe renal toxicity [11]. The kidney selectively accumulates cisplatin and its analogues to a higher degree than other organs, probably through mediated transport [12]. Cisplatin treatment also induces extensive death of cells in the proximal and distal tubules and loop of Henle [13]. High doses of cisplatin produce impairment of kidney and are recognized as the most important dose limiting factor [14]. Mild nephrotoxicty has also been reported with the cisplatin at a dose of 1 mg/kg body wt. [10]. Nephrotoxicity of cisplatin has been reported to be reduced by the administration of antioxidants [15], [16]. At higher doses, cisplatin may be used in combination with antioxidants which might suppress the drug-induced toxic effects, and may help in complete elimination of the parasite from the host reticulo-endothelial system. All these factors led to designing of the present study where we attempted to test the leishmanicidal activity of cisplatin at high dosages of 2.5 and 5 mg/kg b.wt. Since, cisplatin causes nephrotoxicity at higher dosages, we have studied the nephroprotective potential of different antioxidants i.e. vitamin C, vitamin E and silibinin.

## Methods

### Parasite and culture conditions

*Leishmania donovani* promastigotes of strain MHOM/IN/80/Dd8, originally obtained from the London School of Hygiene and Tropical Medicine, London, were used for the present study and maintained *in vitro* at 22±1°C in modified NNN medium by serial subcultures after every 48–72 h.

### Animals

5–6 weeks old inbred BALB/c mice, weighing 20–25 g were obtained from IMTECH and Central Animal House of Panjab University, India. They were fed with water and mouse feed ad libitum.

### Ethics statement

Experiments were carried out according to the guidelines of the Committee for the purpose of Control and Supervision of Experiments on Animals (CPCSEA, Registration No. 45/1999/CPCSEA). The ethical clearance for conducting various experiments mentioned in the study on BALB/c mice was taken from Institutional Animal Ethics Committee (IAEC) of the Panjab University, Chandigarh in its meeting held on 25.08.2008 (Approval No. 1334-50/CAH/3.09.2008).

### Drug

cis-diamminedichloroplatinum (II) dichloride (CP) was purchased from Sigma Aldrich Co., USA and was dissolved in distilled water to get the required concentration of 5 mg/kg body weight (b.wt.) and 2.5 mg/kg body weight (b.wt.). Sodium stibogluconate (SSG) was dissolved in distilled water in water bath at 72°C to get the required concentration of 40 mg/kg b.wt. Vitamin C (Ascorbic acid), vitamin E [(±)-α-Tocopherol] and Silibinin were also purchased from Sigma Aldrich Co., USA. Vitamin C was dissolved in distilled water, Vitamin E was dissolved in corn oil and silibinin was dissolved in saline to get the required concentration of 200 mg/kg b.wt of vitamin C, 100 mg/100 g b.wt. of vitamin E and 200 mg/kg b.wt. of silibinin.

### Infection and drug treatment

#### Infection

Inbred BALB/c mice were infected with the dose of 10^7^ promastigotes of *L. donovani* by intracardiac route [17].

#### Drug treatment

Cisplatin was administered to all groups of animals after 30 post infection days (p.i.d.). All the groups of animals were further divided into two groups (A and B). Group A was treated with cisplatin at a dose of 5 mg/kg b.wt., intraperitoneally for 5 days [18] and group B was treated with cisplatin at a dose of 2.5 mg/kg b.wt., intraperitoneally for 5 days [15]. The group sizes were *n* = 6 for each subgroup (Table 1).

**Table 1:** Various groups used in the study.

Sr. No.	Groups of animals	Dosage
1	Cisplatin+Silibinin	200 mg/kg b.wt. of silibinin, given intraperitoneally.
2	Cisplatin+VitaminC+VitaminE	100 mg/100 g b.wt. of vitamin E and 200 mg/kg b.wt. of Vitamin C, both given orally.
3	Cisplatin+Silibinin+Vitamin C+Vitamin E	200 mg/kg b.wt. of silibinin given intraperitoneally. 100 mg/100 g b.wt. of vitamin E and 200 mg/kg b.wt. of Vitamin C, both given orally.
4	Sodium stibogluconate (SSG) (Positive control)	40 mg/kg b.wt. for 5 days
5	Infected control	10^7^ promastigotes of *L. donovani* by intracardiac route.
6	Normal control	Saline treated

#### Assessment of Infection

After the completion of five days of treatment, 6 mice from each group were sacrificed after 1, 15, 30 post treatment days (p.t.d.) along with infected and normal controls. Parasite load was assessed in the impression smears of liver in terms of Leishman Donovan Units by the method of Bradley and Kirkley [19].

#### Delayed type hypersensitivity (DTH) response

All groups of mice were challenged in the right foot pad with a subcutaneous injection of leishmanin. [For preparing leishmanin, promastigotes in the stationary phase of growth were harvested from modified NNN medium and washed thrice with PBS (Phosphate buffer saline). The final pellet was then suspended in 5 ml of 0.5% phenol in sterile PBS and kept at room temperature for 10 min. The phenol was then removed and the final concentration was adjusted to 2×10^8^ promastigotes per ml]. After 48 hr, the thickness of the right and left foot pads were measured using vernier calliper. The percentage increase in the thickness of the right foot pad as compared to the left foot pad of mice was calculated [20].

#### Assessment of antibody response

The humoral immune response induced by the antigens was evaluated by measuring total specific IgG by indirect ELISA from serum samples collected from different groups of animals by using commercially available kits (Bangalore Genei, Bangalore, India).

#### ELISA for parasite-specific IgG1 and IgG2a isotypes

The specific serum immunoglobulin G (IgG) isotype antibody response was measured by conventional ELISA [20] by using commercially available kits (Bangalore Genei, Bangalore, India).

#### Determination of drug-induced cytokine production

The lymphocytes from spleens of infected and drug treated mice of different groups were cultured in 24 well plates in RPMI-1640 medium containing 20 mM NaHCO_3_, 10 mM HEPES, 10 U/ml of penicillin, 100 µg/ml streptomycin, 2 mM L-glutamine and 10% FCS. Cells were stimulated with 50 µg/ml of the crude antigen and incubated at 37°C for 72 h and supernatant of all cultures was collected and stored at −20°C till further use. These were subsequently used for the determination of cytokine levels using commercially available kits from Bender Med Systems, Austria (IL-4 and IL-10) and Diaclone, France (IFN-γ and IL-2) according to manufacturer's instructions.

#### Evaluation of hematological parameters

Hemoglobin (Hb) estimation was done by Sahli's hemometer (Marienfeld Lab. Glassware, Germany) and total leukocyte count (TLC) estimation was done by the method of Khynriam and Prasad [21].

### Evaluation of biochemical parameters

#### Liver Function Tests

The estimation of Alkaline phosphatase (ALP), Acid phosphatase (ACP), Lactate dehydrogenase (LDH), Serum Glutamate Oxaloacetate Transaminase (SGOT), Serum Glutamate Pyruvate Transaminase (SGPT) was done in serum samples by using commercially available kits (Reckon Diagnostics Pvt. Ltd Baroda, India; Span Diagnostic Ltd. and Kinetik Koncepts, Gurgaon, India).).

#### Kidney Function Tests

The estimation of Urea, blood urea nitrogen (BUN), Creatinine, Uric acid and the electrolytes like Na^+^, K^+^, Mg^++^, Cl^−^, Ca^++^ and PO_4_ was done in serum samples using commercially available kits (Reckon Diagnostics Pvt. Ltd Baroda, India; Transasia Bio-medical Ltd. India; Coral Clinical Systems, Goa, India and M/S Excel Diagnostics Pvt. Ltd. India).

#### Mortality rate

Mortality rate was calculated in order to estimate the overall mortality among different group of animals.

#### Statistical analysis

All the experiments were performed three times independently. All data comparisons were tested for significance by using one-way ANOVA; P values of <0.05 and <0.001 were considered significant. Results were expressed as mean±S.D. of one of three independent experiments.

## Results

### Hepatic parasite load

**Figure 1:**
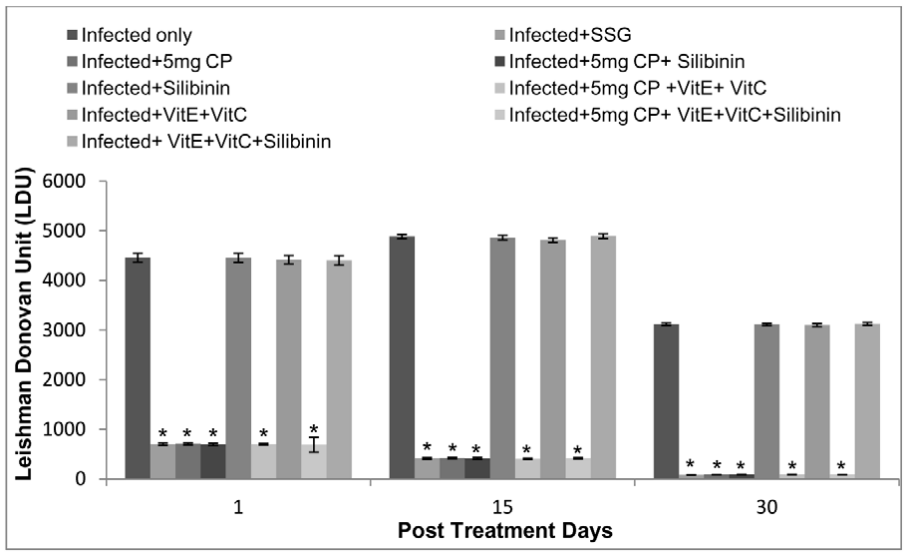
Parasite load in terms of LDU in different groups of animals. The data are presented as mean±S.D. of six mice per group. * -p value: Infected only vs Infected+SSG/Infected+5 mg CP/Infected+5 mg CP+Silibinin/Infected+5 mg CP+VitE+VitC/Infected+5 mg CP+VitE+VitC+Silibinin.* - p<0.001. No significant difference was found in groups: Infected+5 mg CP vs Infected+5 mg CP+Silibinin/Infected+5 mg CP+VitE+VitC/Infected+5 mg CP+VitE+VitC+Silibinin and Infected only vs Infected+Silibinin/Infected+VitE+VitC/Infected+VitE+VitC+Silibinin. LDU- Leishman Donovan Units.

Mice treated with cisplatin showed significant reduction in the parasite load as compared to infected untreated controls. Cisplatin at the dosage of 5 mg/kg b.wt. showed significantly lesser parasite burden as compared to those treated with 2.5 mg/kg b.wt. of cisplatin. The reduction in parasite burden in cisplatin treated groups was observed to be 97% (5 mg/kg b.wt.) and 91% (2.5 mg/kg b.wt.) on 30 p.t.d. The percent decrease in parasite load was found to be in the range of 84–97% in animals treated with cisplatin along with different antioxidants. Similar trend was observed in mice treated with 2.5 mg/kg b.wt. of cisplatin. Results were also comparable with the group of infected animals treated with SSG (positive control), where reduction in parasite burden was found to be in the range of 84–97% on 1, 15 and 30 p.t.d. (Fig. 1).

### Delayed type hypersensitivity response

**Figure 2:**
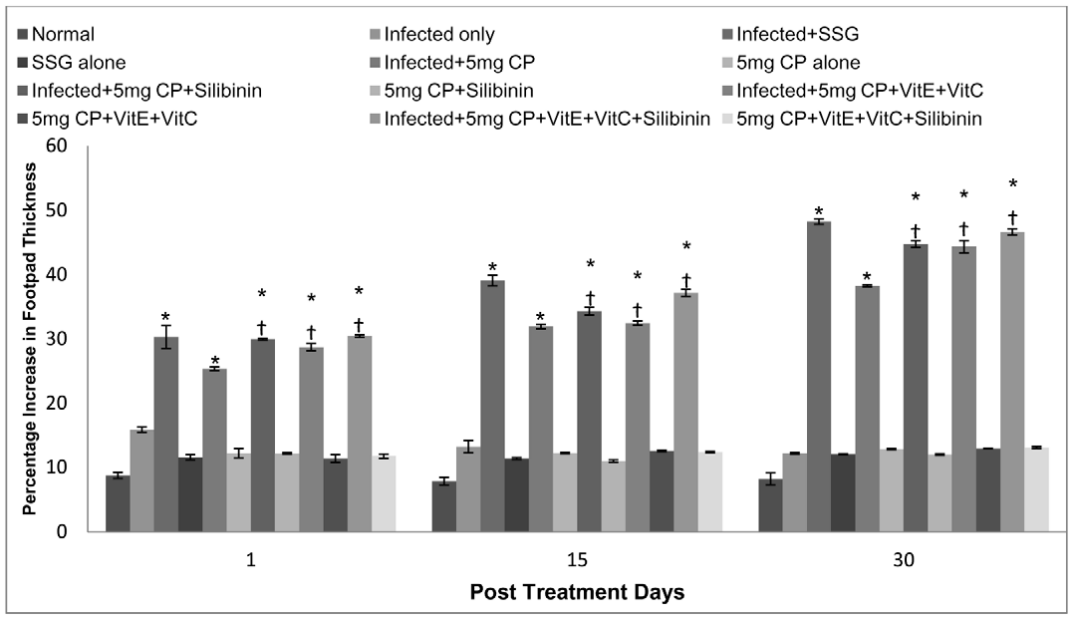
Percentage increase in footpad thickness (DTH response) in different groups of animals. The data are presented as mean±S.D. of six mice per group. * -p value: Infected only vs Infected+SSG/Infected+5 mg CP/Infected+5 mg CP+Silibinin/Infected+5 mg CP+VitE+VitC/Infected+5 mg CP+VitE+VitC+Silibinin. † -p value: Infected+5 mg CP vs. Infected+5 mg CP+Silibinin/Infected+5 mg CP+VitE+VitC/Infected+5 mg CP+VitE+VitC+Silibinin. *,†-p<0.001.

A profound delayed type hypersensitivity response was induced by cisplatin treated *L. donovani* infected animals, suggesting the generation of cell-mediated immune responses. The percentage increase in footpad thickness in the infected animals treated with 5 mg/kg b.wt. of cisplatin was found to be significantly higher than those treated with 2.5 mg/kg b.wt. of the drug. In infected animals where antioxidants were given along with cisplatin at the dosage of 5 mg/kg b.wt., the DTH response was significantly increased from 1 to 30 p.t.d. The increase in DTH response varies from 28–46% in animals treated with cisplatin along with antioxidants. Furthermore, treatment with cisplatin (2.5 mg/kg bwt.) in combination with different antioxidants, significantly increased the DTH response on 30 p.t.d. as compared to infected controls. When cisplatin treated animals were compared with SSG treated animals then the increase was found to be comparable (Fig. 2).

### Assessment of antibody response

**Figure 3:**
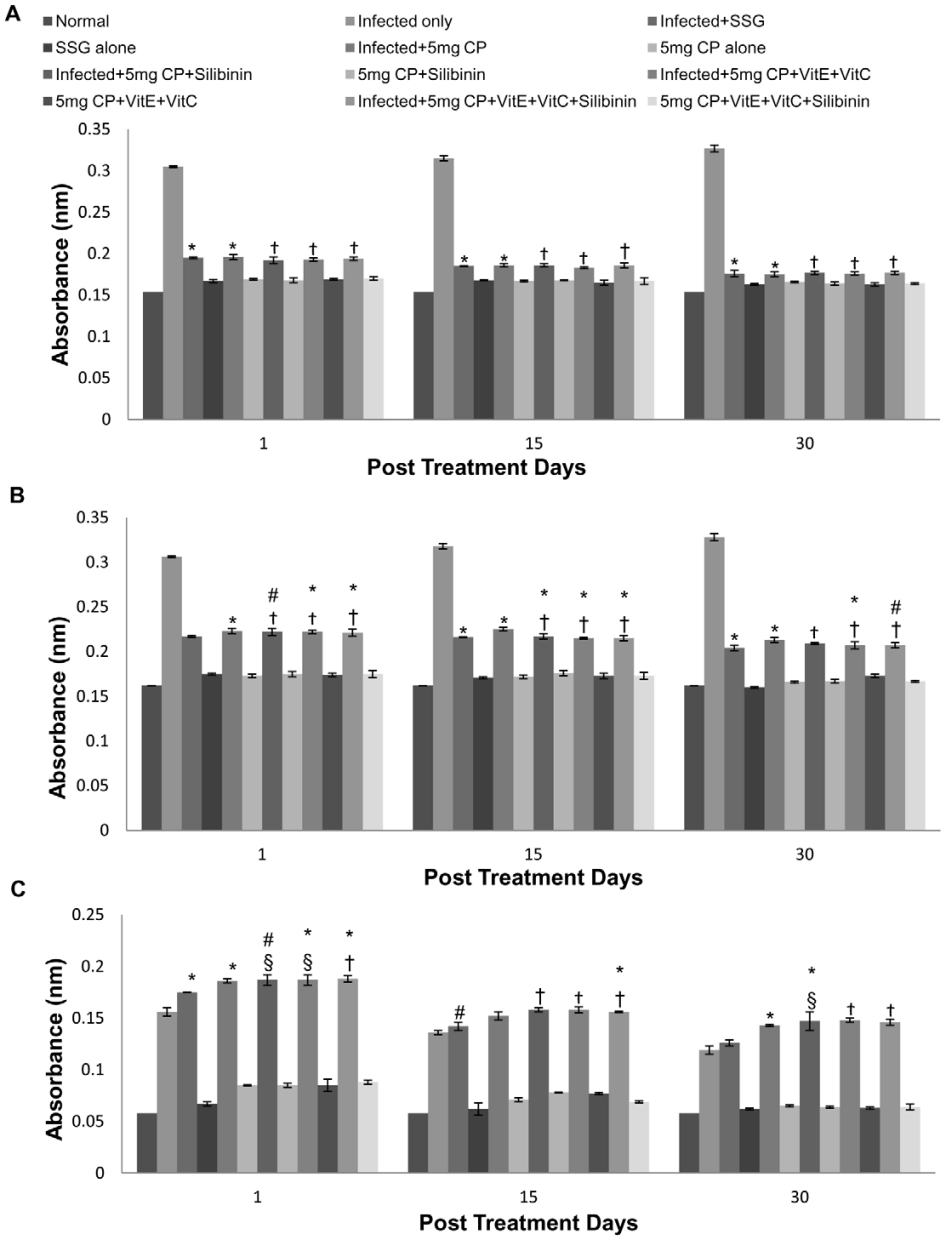
Levels of *Leishmania*-specific IgG antibodies in serum samples of different groups of animals. A -IgG, B -IgG1 and C -IgG2a. The data are presented as mean±S.D. of six mice per group. *,# -p value: Infected only vs Infected+SSG/Infected+5 mg CP/Infected+5 mg CP+Silibinin/Infected+5 mg CP+VitE+VitC/Infected+5 mg CP+VitE+VitC+Silibinin. †,§ -p value: Infected+5 mg CP vs. Infected+5 mg CP+Silibinin/Infected+5 mg CP+VitE+VitC/Infected+5 mg CP+VitE+VitC+Silibinin. *,†-p<0.001; #,§-p<0.05.

The IgG levels were found to be highest in the infected untreated controls as compared to the infected cisplatin treated animals. With increase in post treatment days, the IgG antibody response in infected mice treated with cisplatin (5 mg/kg b.wt. and 2.5 mg/kg b.wt.) was observed to be significantly lower as compared to control animals and the maximum antibody response was produced in infected untreated animals. IgG levels in the infected animals treated with 5 mg/kg b.wt. of cisplatin were found to be significantly lower than those treated with 2.5 mg/kg b.wt. of the drug. When cisplatin treated animals were compared with SSG treated animals then the decrease in antibody titre was found to be comparable (Fig. 3A).

### ELISA for parasite-specific IgG1 and IgG2a isotypes

IgG1 and IgG2a antibody responses were also evaluated by ELISA using specific anti-mouse isotype antibodies. Similarly, in addition to IgG levels, decreased IgG1 levels were found in infected cisplatin treated animals as compared to infected untreated controls.

Treatment of infected animals with cisplatin (5 mg/kg b.wt. and 2.5 mg/kg b.wt.) significantly increased the IgG2a antibody titre on 30 p.t.d. as compared to infected animals. Treatment of animals with cisplatin led to a sudden increase in the IgG2a levels on 1 p.t.d. and further decreased on 30 p.t.d. but was still higher than infected controls. The increase was more pronounced in infected animals treated with cisplatin at a dosage of 5 mg/kg b.wt. as compared to infected animals treated with cisplatin at dosage of 2.5 mg/kg b.wt. When antioxidants were supplemented along with cisplatin at the dosage of 5 mg/kg b.wt., the IgG2a antibody titre was found to be higher than the infected controls. This increase in IgG2a antibody titre varies from 0.188±0.003–0.146±0.003 in cisplatin+vitC+vitE+silibinin, 0.187±0.005–0.148±0.002 in cisplatin+vitC+vitE and 0.187±0.0.005–0.147±0.0.009 in cisplatin+silibinin treated animals on 30 p.t.d. Similar trend was observed in mice treated with 2.5 mg/kg bwt. of cisplatin. When cisplatin treated animals were compared with SSG treated animals then the increase in antibody titres was found to be comparable (Fig. 3B and 3C).

### Determination of drug-induced cytokine production

**Figure 4:**
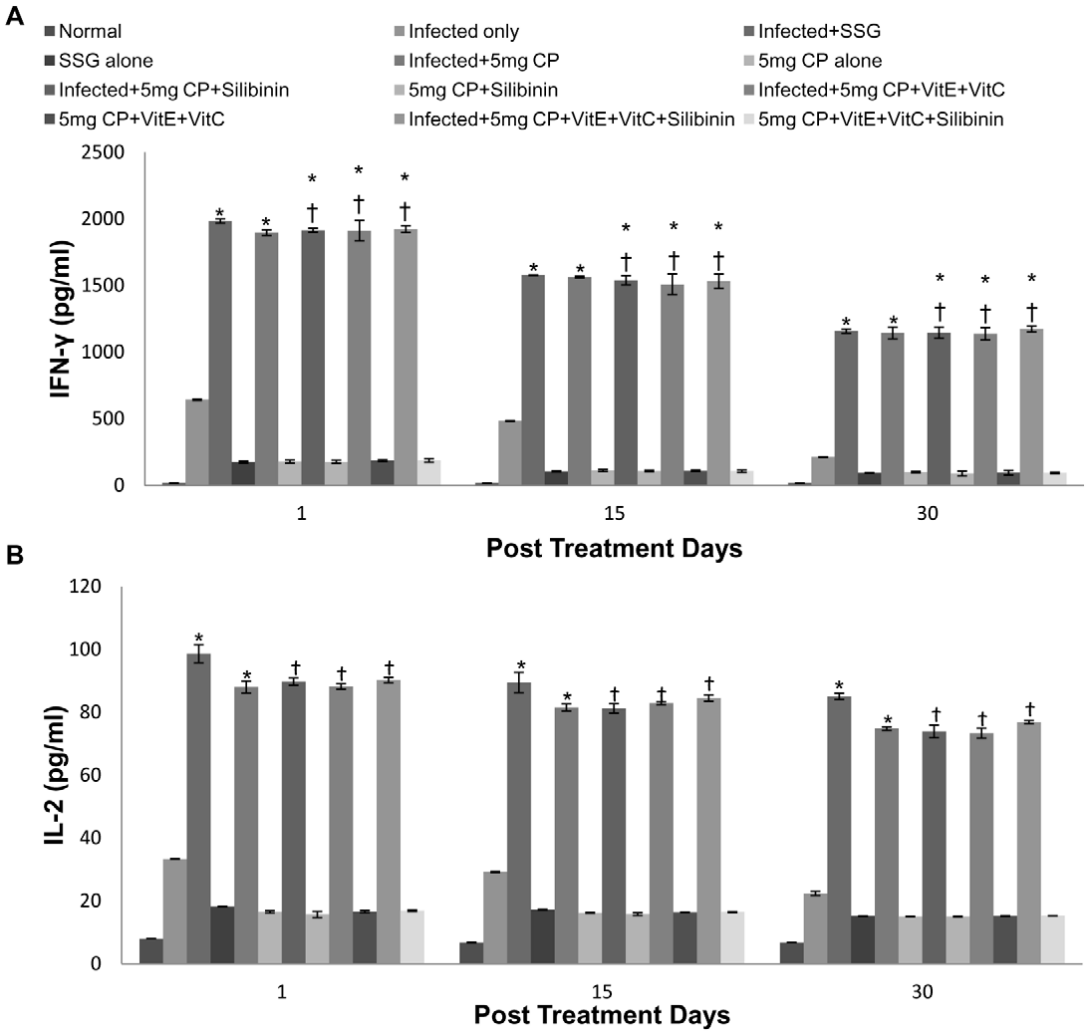
Cytokine levels in culture supernatants of spleen cells of different groups of animals. A- IFN-γ, B-IL-2. The data are presented as mean±S.D. of six mice per group. * -p value: Infected only vs Infected+SSG/Infected+5 mg CP/Infected+5 mg CP+Silibinin/Infected+5 mg CP+VitE+VitC/Infected+5 mg CP+VitE+VitC+Silibinin. † -p value: Infected+5 mg CP vs. Infected+5 mg CP+Silibinin/Infected+5 mg CP+VitE+VitC/Infected+5 mg CP+VitE+VitC+Silibinin. *,†-p<0.001.

The cytokine responses (IFN-γ, IL-2, IL-4 and IL-10) in supernatants of spleen cells, cultured in the presence of crude antigen (50 µg/ml), were analyzed for different groups of animals. Th1-specific cytokines, that is, IFN-γ and IL-2 levels were significantly greater in infected mice treated with cisplatin (5 mg/kg b.wt. and 2.5 mg/kg b.wt.) as compared to infected untreated animals and uninfected treated mice. The levels of IFN-γ and IL-2 decreased from 1 p.t.d. to 30 p.t.d., however they were still higher than infected controls. The levels of these cytokines were comparable when cisplatin treated animals were compared with cisplatin and antioxidants treated animals. Also, when the cisplatin treated animals were compared with SSG treated animals then the increase in IFN-γ and IL-2 levels were found to be comparable (Fig. 4A and 4B).

**Figure 5:**
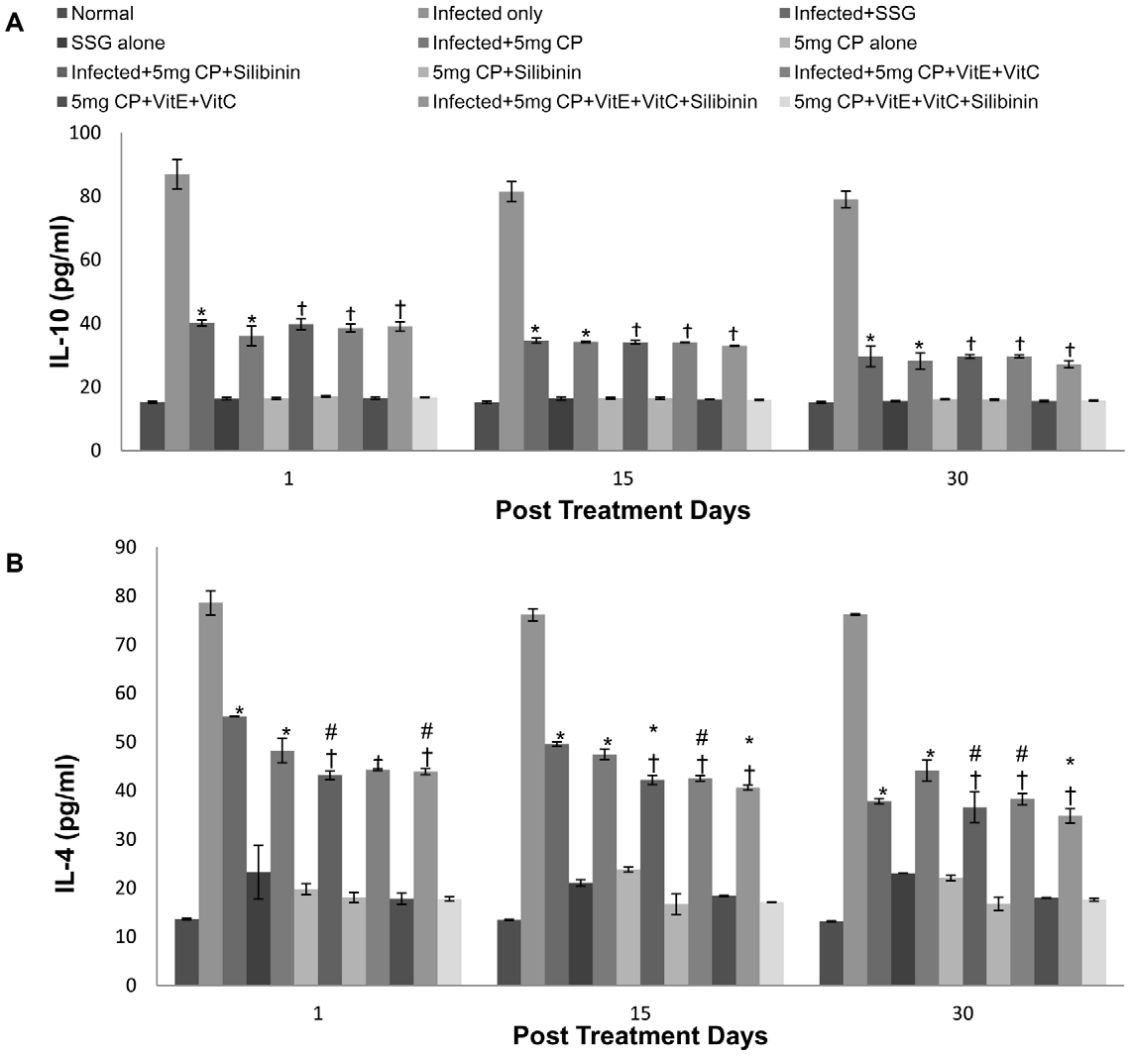
Cytokine levels in culture supernatants of spleen cells of different groups of animals. A- IL-10, B- IL-4. The data are presented as mean±S.D. of six mice per group. *,# -p value: Infected only vs Infected+SSG/Infected+5 mg CP/Infected+5 mg CP+Silibinin/Infected+5 mg CP+VitE+VitC/Infected+5 mg CP+VitE+VitC+Silibinin. † -p value: Infected+5 mg CP vs. Infected+5 mg CP+Silibinin/Infected+5 mg CP+VitE+VitC/Infected+5 mg CP+VitE+VitC+Silibinin. *,†-p<0.001; #-p<0.05.

The levels of Th2-regulated cytokines, IL-4 and IL-10, were minimum in the infected animals treated with cisplatin. Spleen cells from infected mice, however, produced much more IL-4 than the cisplatin treated groups. This effect of cisplatin in down-regulating IL-4 and IL-10 production was seen in almost all the animals treated along with different antioxidants on different post treatment days. The levels of IL-10 and IL-4 produced by splenocytes of infected and cisplatin treated mice were comparable to that induced by SSG treatment. (Fig. 5A and 5B).

### Hematological investigations

**Figure 6:**
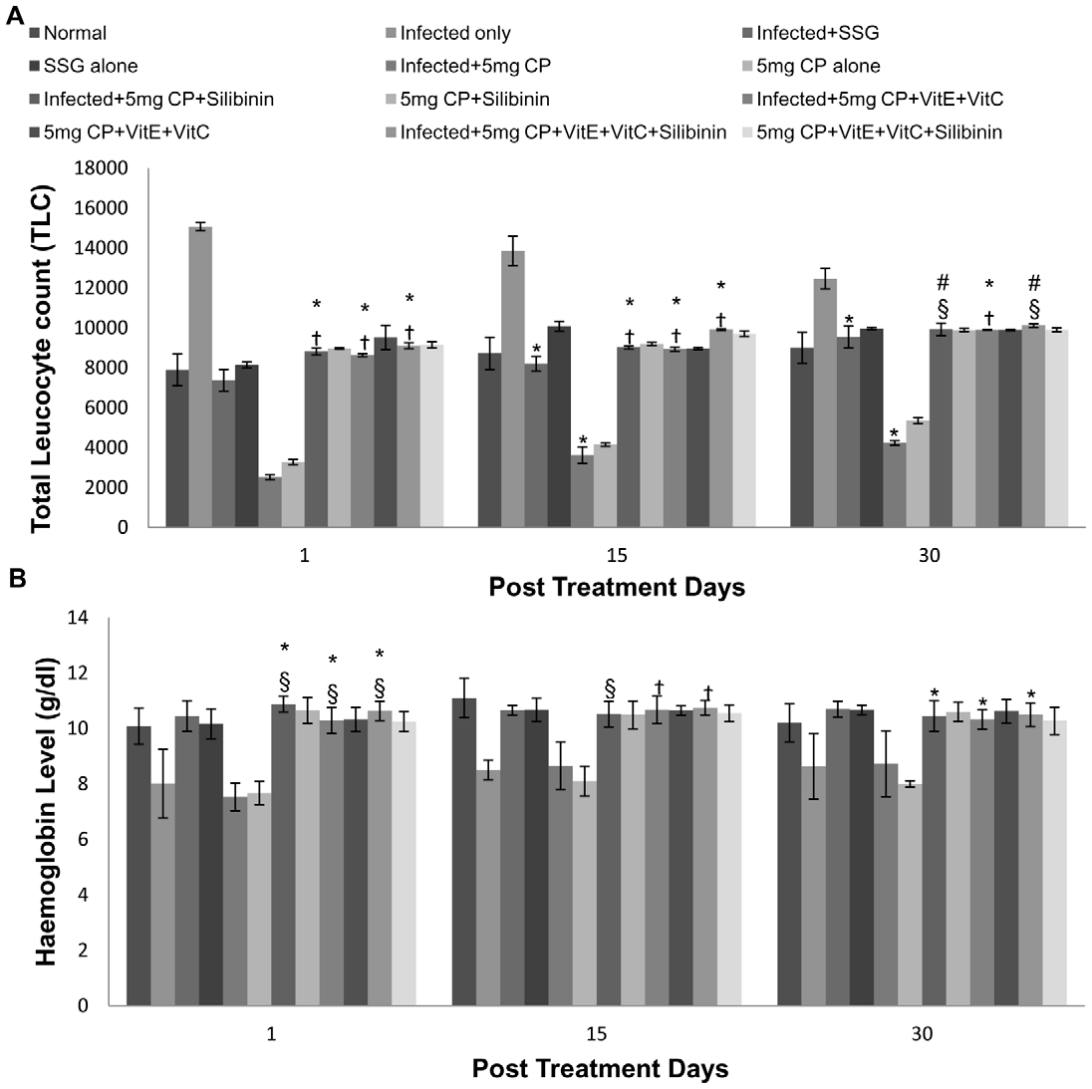
Total leucocyte count and hemoglobin levels in different groups of animals. A- Total Leukocyte Count, B- Hemoglobin levels. The data are presented as mean±S.D. of six mice per group. *,# -p value: Infected only vs Infected+SSG/Infected+5 mg CP/Infected+5 mg CP+Silibinin/Infected+5 mg CP+VitE+VitC/Infected+5 mg CP+VitE+VitC+Silibinin. †,§ -p value: Infected+5 mg CP vs. Infected+5 mg CP+Silibinin/Infected+5 mg CP+VitE+VitC/Infected+5 mg CP+VitE+VitC+Silibinin. *,†-p<0.001; #,§-p<0.05.

A decrease in hemoglobin levels were observed in infected and uninfected cisplatin (5 mg/kg b.wt. and 2.5 mg/kg b.wt.) treated animals as compared to normal control animals. When antioxidants were given along with the drug in infected animals, hemoglobin levels were found to be in normal range of 8–10 g/dl. Leucopenia was observed in infected and uninfected cisplatin (5 mg/kg b.wt. and 2.5 mg/kg b.wt.) treated animals while leucocytosis was observed in infected untreated animals. TLC was found to be in normal range of 7000–12000/mm^3^ when antioxidants were supplemented along with cisplatin. When compared with SSG, the results were found to be comparable (Fig. 6A and 6B).

### Liver function tests

**Figure 7:**
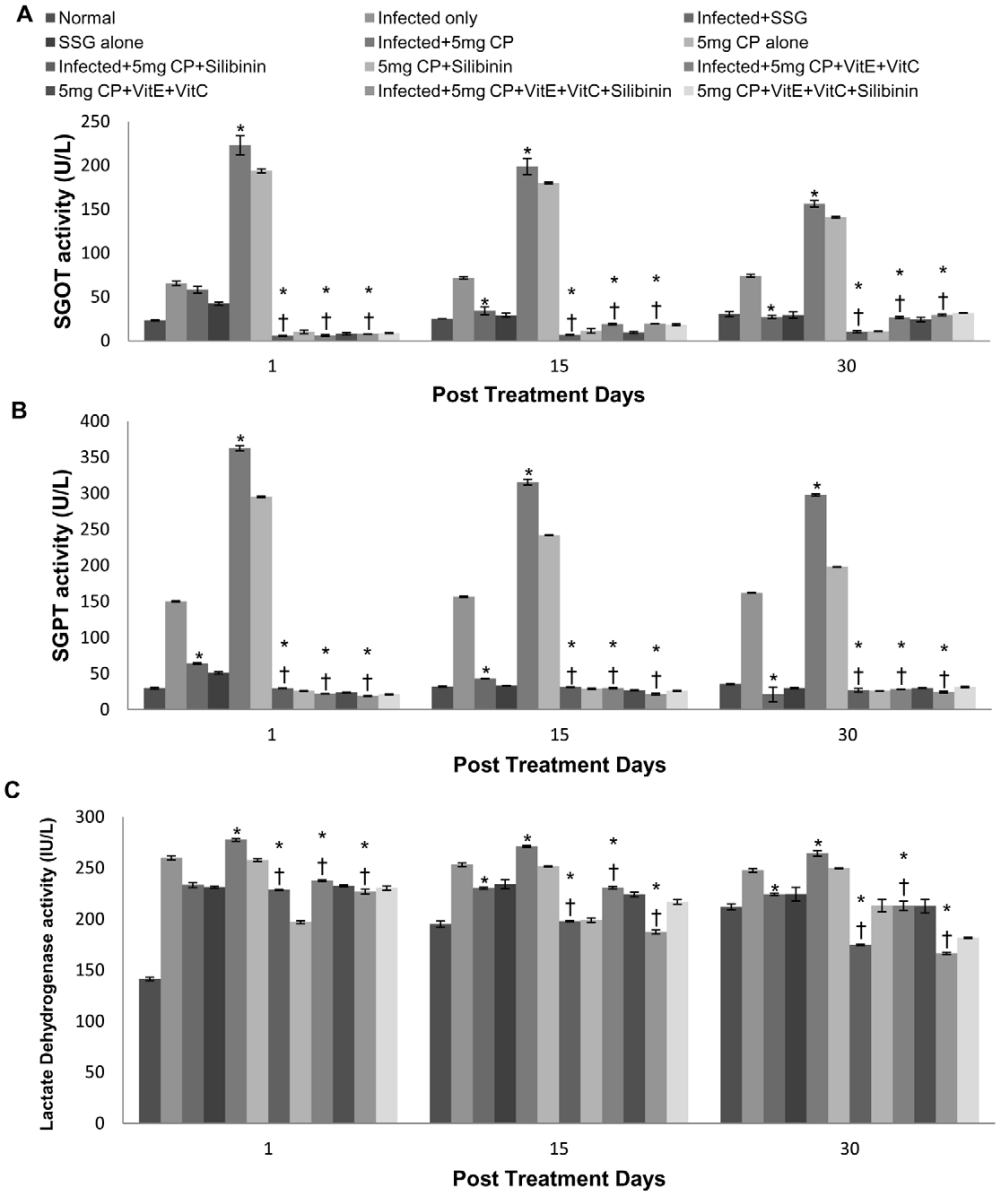
SGOT, SGPT and LDH activity in serum samples of different groups of animals. A- SGOT, B-SGPT, C-LDH. The data are presented as mean±S.D. of six mice per group.* -p value: Infected only vs Infected+SSG/Infected+5 mg CP/Infected+5 mg CP+Silibinin/Infected+5 mg CP+VitE+VitC/Infected+5 mg CP+VitE+VitC+Silibinin. † -p value: Infected+5 mg CP vs. Infected+5 mg CP+Silibinin/Infected+5 mg CP+VitE+VitC/Infected+5 mg CP+VitE+VitC+Silibinin. *,†-p<0.001.

Quantitative estimation of SGOT and SGPT activity revealed maximum activity in infected mice treated with cisplatin followed by uninfected cisplatin treated mice and then infected untreated mice. Enzyme activity in mice treated with 5 mg/kg b.wt. of cisplatin was found to be maximum in comparison to those treated with 2.5 mg/kg b.wt. of the drug and thus showed a sharp decline from 1 to 30 p.t.d. when antioxidants were supplemented along with the cisplatin at the dosage of 5 mg/kg b.wt. The percent decrease in SGOT level was found to be 96–80% in cisplatin+vitC+vitE+silibinin, 97–82% in cisplatin+vitC+vitE and 97–93% in cisplatin+silibinin treated animals on 30 p.t.d. (Fig. 7A).

The percent decrease in SGPT level was found to be 94–91% in cisplatin+vitC+vitE+silibinin, 93–90% in cisplatin+vitC+vitE and 91.86–91.98% in cisplatin+silibinin treated animals on 30 p.t.d. as compared to infected cisplatin treated mice (Fig. 7B).

Similarly, SGOT and SGPT levels were found to be significantly reduced when antioxidants were given with cisplatin at the dosage of 2.5 mg/kg bwt. When compared with SSG, the results were found to be comparable.

The alkaline phosphatase and acid phosphatase activity was found to be in normal range of 4–11 KA units and 0 to 0.6 U/L respectively in all groups of mice.

The activity of lactate dehydrogenase was found to be maximum in infected mice treated with cisplatin followed by infected untreated mice and uninfected untreated mice. When antioxidants were supplemented along with cisplatin, the significantly reduced levels of LDH were found on 1 p.t.d. as compared to cisplatin treated mice and normal range of 114–240 IU/L was observed on all post treatment days. When compared with SSG, the results were found to be comparable (Fig. 7C).

### Kidney function test

**Figure 8:**
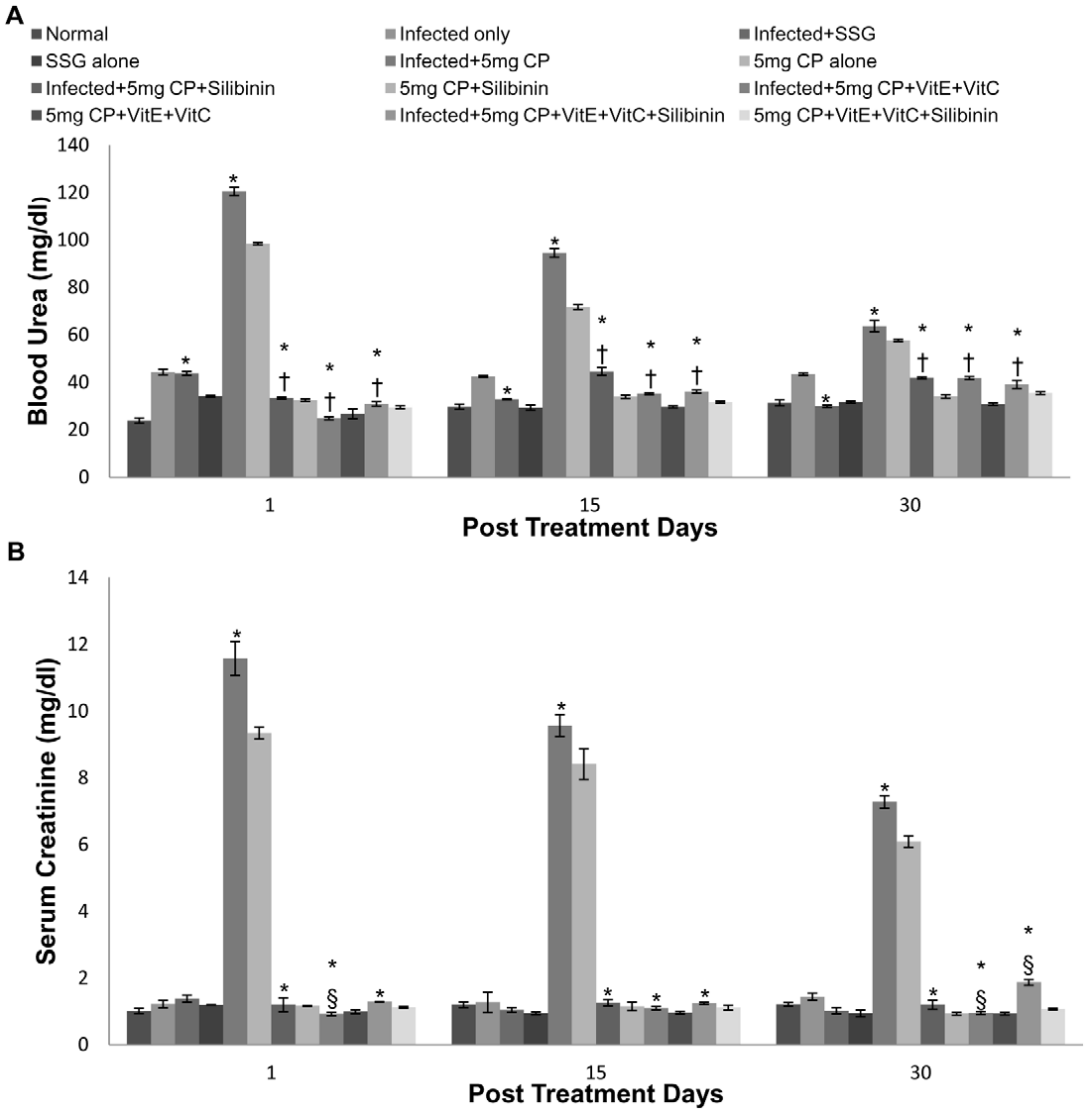
Blood urea and creatinine levels in serum samples of different groups of animals. A- Blood Urea, B- Creatinine. The data are presented as mean±S.D. of six mice per group. * -p value: Infected only vs Infected+SSG/Infected+5 mg CP/Infected+5 mg CP+Silibinin/Infected+5 mg CP+VitE+VitC/Infected+5 mg CP+VitE+VitC+Silibinin. †,§ -p value: Infected+5 mg CP vs. Infected+5 mg CP+Silibinin/Infected+5 mg CP+VitE+VitC/Infected+5 mg CP+VitE+VitC+Silibinin. *,†-p<0.001; §-p<0.05.

Treatment of infected and uninfected mice with cisplatin (5 mg/kg bwt and 2.5 mg/kg bwt) led to a sudden increase in the concentration of blood urea, BUN, uric acid and creatinine. The increase was more pronounced in mice treated at the dosage of 5 mg/kg b.wt. as compared to cisplatin at the dosage of 2.5 mg/kg b.wt. To reduce the nephrotoxicity induced by cisplatin, antioxidants (vitamin C, vitamin E and silibinin) were supplemented along with cisplatin. The levels of serum urea, BUN, uric acid and creatinine were found to be within the normal range of 10–45 mg/dl, 5–21 mg/dl, 3–6.7 mg/dl and 0.85–1.35 mg/dl respectively in animals treated with cisplatin along with antioxidants. The results were quite comparable to the SSG treated mice where normal levels were found on different post treatment days (Fig. 8A and 8B).

**Figure 9:**
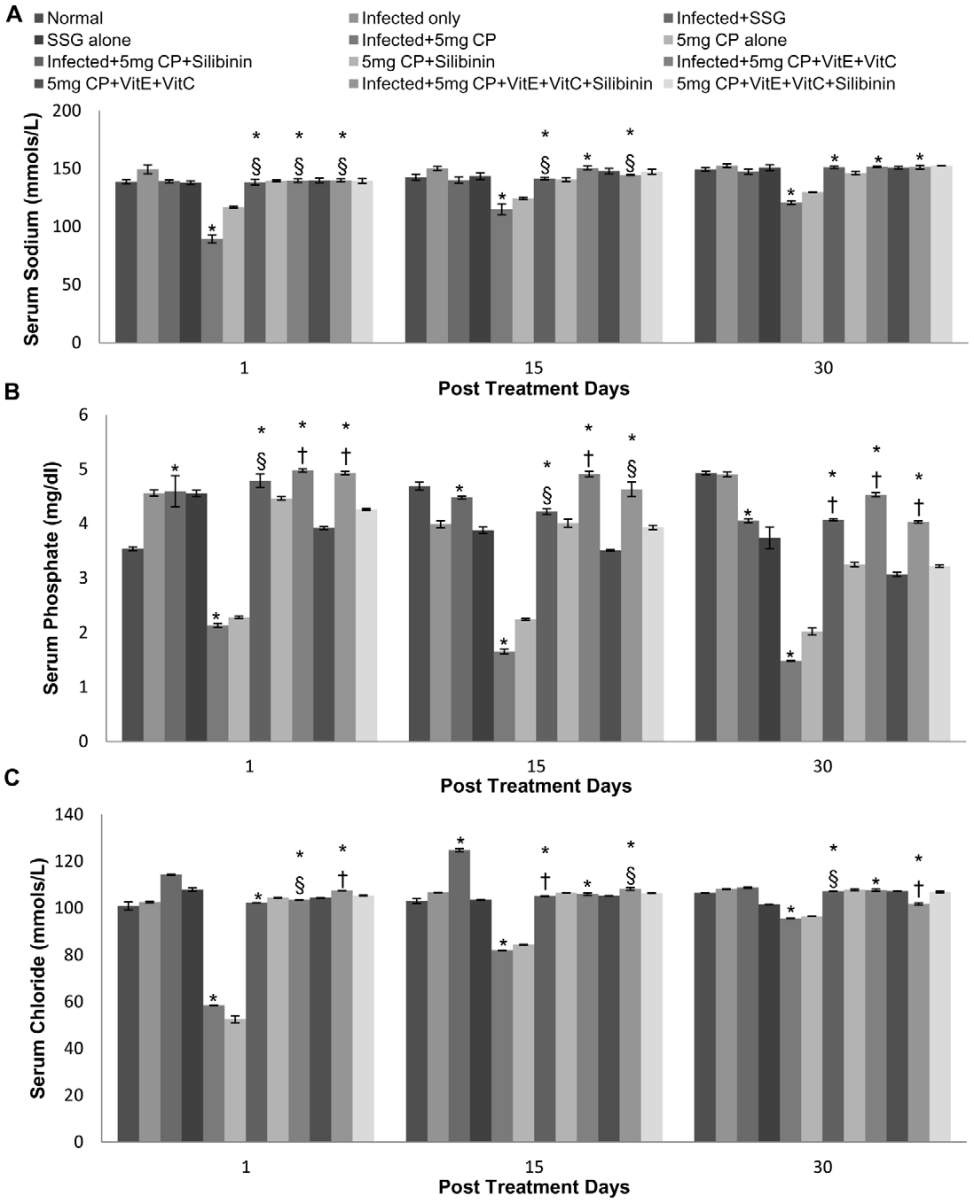
Sodium, phosphate and chloride levels in serum samples of different groups of animals. A- Sodium, B- Phosphate, C- Chloride. The data are presented as mean±S.D. of six mice per group.* -p value: Infected only vs Infected+SSG/Infected+5 mg CP/Infected+5 mg CP+Silibinin/Infected+5 mg CP+VitE+VitC/Infected+5 mg CP+VitE+VitC+Silibinin. †,§ -p value: Infected+5 mg CP vs. Infected+5 mg CP+Silibinin/Infected+5 mg CP+VitE+VitC/Infected+5 mg CP+VitE+VitC+Silibinin. *,†-p<0.001; §-p<0.05.

**Figure 10:**
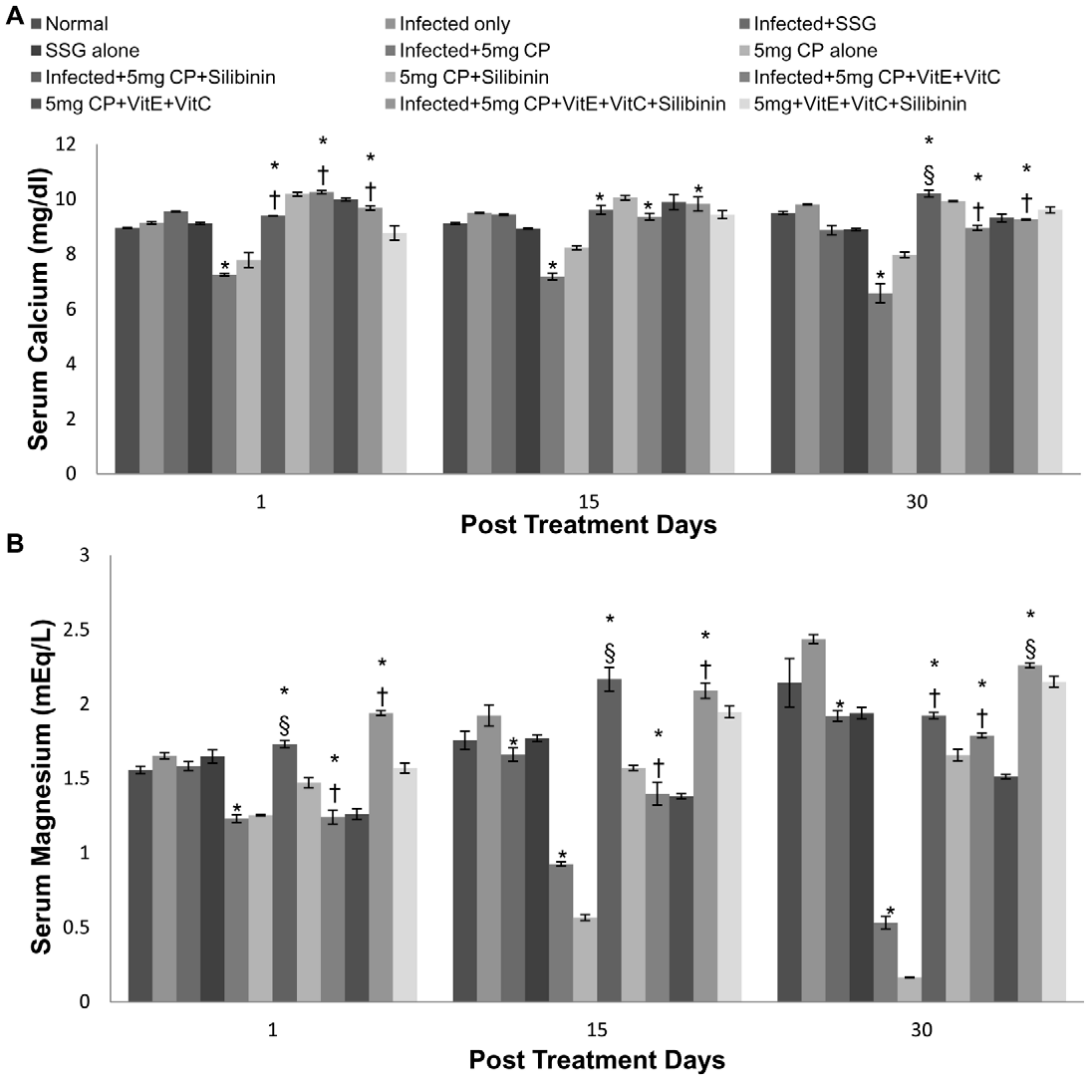
Calcium and magnesium levels in serum samples of different groups of animals. A- Calcium, B- Magnesium. The data are presented as mean±S.D. of six mice per group. * -p value: Infected only vs Infected+SSG/Infected+5 mg CP/Infected+5 mg CP+Silibinin/Infected+5 mg CP+VitE+VitC/Infected+5 mg CP+VitE+VitC+Silibinin. †,§ -p value: Infected+5 mg CP vs. Infected+5 mg CP+Silibinin/Infected+5 mg CP+VitE+VitC/Infected+5 mg CP+VitE+VitC+Silibinin. *,†-p<0.001; §-p<0.05.

A significant decrease was found in electrolyte levels when infected animals were treated with cisplatin and leads to hyponatremia, hypomagnesemia, hypocalcemia, hypokalemia, hypochloremia and hypophosphatemia. The decrease in electrolytes was more pronounced in mice treated with 5 mg/kg b.wt. of cisplatin in comparison to those treated with 2.5 mg/kg b.wt. of cisplatin. When the antioxidants were supplemented to reduce the nephrotoxicity, the normal electrolyte levels were attained and the serum sodium, potassium, phosphorus, chloride, calcium and magnesium concentration was found to be within the range of 135 to 155 mmols/l, 3.6 to 5.5 mmols/l, 2.5–5 mg/dl, 98–109 mmols/l, 8.7 to 10.5 mg/dl and 1.3 to 2.5 mg/dl respectively (Fig. 9A, 9B, 9C and Fig. 10A, 10B).

### Mortality rate

The death rate was maximum in animals treated with cisplatin (5 mg/kg bwt and 2.5 mg/kg bwt). This increased from 30–85% and 25–75% in animals treated with cisplatin at the dosage of 5 mg/kg bwt. and 2.5 mg/kg bwt. on different post treatment days. When antioxidants were given along with the drug in infected animals, death rate was found to be 0%. The results were found to be comparable to SSG treated animals where death rate was found to be 0% on 30 p.t.d.

## Discussion

The results of the present study demonstrated the antileishmanial efficacy of high doses of cisplatin. In addition treatment of *L. donovani* infected animals with cisplatin along with antioxidants (vitamin C, vitamin E and silibinin) ameliorated the nephrotoxicity caused by administration of high dosage of cisplatin. The drug-induced protective immune responses were associated with a reduction in parasite burden as assessed by LDU in liver. It has been observed that mice treated with higher dosage of cisplatin (5 mg/kg b.wt.) showed better results by reducing the parasite load by 97% as compared to low dosage (2.5 mg/kg b.wt.) which proves its efficacy against the amastigote stage of *L. donovani*. These findings correlate with the earlier studies from our laboratory where the drug was found to be more effective at a concentration of 1 mg/kg b.wt. as compared to 0.5 mg/kg b.wt. [10]. SSG at a dosage of 40 mg/kg/day for 5 days intraperitoneally resulted in reduction of parasite burden by 97% from liver. Similar observations have been made by Trotter et al [22] who found that 47 mg/kg/day of pentostam for 5 days cured 90% of infection in NMRI mice. Our studies are also consistent with several previous studies which confirmed that SSG therapy cured more than 95% of patients in Bihar [23]. In Pakistan, Nepal and Sudan, cure rates in the range of 98%–100% have been reported [24]–[26] The effectiveness of the newly developed antineoplastic drug, cisplatin (cis-diamminedichloroplatinum II; CDDP) was comparable with that of SSG as treatment with cisplatin resulted in reduction of parasite load to a similar extent.

Our studies were in accordance with the findings of Geib et al [27] where irinotecan/cisplatin treatment related deaths were observed in 5 cases (19%), mainly due to infectious complications due to excessive toxicity. Cisplatin should be used in combination with antioxidants to overcome the side effects induced by cisplatin and to increase the survival rate of animals. Our study has shown that after antioxidant supplementation along with cisplatin, no death was reported. Several cellular studies, animal and human studies [28]–[31] have demonstrated that vitamins A, E, C, and K, as well as beta-carotene and selenium—as single agents or in combination—all protect against the toxicity of adriamycin and actually enhance its cancer-killing effects and increase the survival rate.

Delayed type hypersensitivity (DTH) is an immunologic response that has been frequently used as a correlate for protection against or sensitization to *Leishmania* antigen in humans and experimental models of *Leishmania* infection [32]. The results demonstrate a positive correlation between enhanced DTH responses and reduced parasite load demonstrating the generation of Th1 type of protective immune responses [33] generated by cisplatin. This response was found to be more pronounced in mice treated with 5 mg/kg b.wt. as compared to those treated with 2.5 mg/kg b.wt. of cisplatin since maximum reduction in parasite burden occurs after treatment with high dosage of cisplatin. We have earlier reported increased DTH responses in cisplatin treated animals, when two different doses (1 mg/kg b.wt. and 0.5 mg/kg b.wt.) of cisplatin were tested. Higher dose revealed enhanced DTH response as compared to a lower dose of cisplatin [10]. The positive DTH response, which is an indicator of development of cell-mediated immune responses, also develops after treatment with the drug indicating thereby that this drug not only brought about reduction in parasite load but also helped in regaining the cell-mediated immune responses which is very important for complete recovery. None of the two doses of the drug caused depression of DTH response, thereby indicating that drug treatment helped in reversal of immunosuppression caused by the parasite.

Since, IgG2a and IgG1 kinetics indirectly reflect the Th1/Th2 responses, the relative production of these isotypes are used as a marker for the induction of Th1-like and Th2-like immune responses. The analysis of IgG isotypes disclosed a dichotomous response to visceral infection. Treated animals produced low levels of IgG and IgG1 in comparison to the infected controls but IgG2a levels were reported to be slightly higher. A successful cure results when levels of IgG2a increase with low levels of IgG1 in treated groups driving the immune response towards Th1 type. The results demonstrate a positive correlation of low IgG1 with high cell mediated immune response, in terms of DTH and vice versa.

There is a correlation between the clinical outcome of the infection and the cytokine response profile. Control of visceral leishmaniasis in mice is believed to require IFN-γ, produced by spleen cells, which drives the immune response towards a Th1 phenotype by IL-2 [34]. To study the type of immune response generated in the treated animals, the cytokine levels were estimated in the splenic lymphocyte cultures of all groups of mice. Our results suggest that treatment with cisplatin preferentially induces a type 1 immune response which resulted in significant protection in mice against *L. donovani* infection. The infected and cisplatin treated animals showed the maximum concentration of Th1-specific cytokines, IFN-γ and IL-2 and least concentration of Th2-specific cytokines, IL-4 and IL-10 pointing towards the potential of the drug to generate protective immune response against *L. donovani*. High levels of IFN-γ were also reported in the patients exposed to whole cell extracts of the antigen [35]. Cisplatin (DDP) is one of the conventional anticancer agents endowed with immunomodulating features [36]. When the dose of cisplatin was increased to 5 mg/kg b.wt., all mice treated with this dosage of drug showed increased production of Th1 specific cytokines suggesting the generation of Th1 type of immune response and the decrease in parasite load showed protective nature of therapy. The increase was also found to be prominent at low dosage (2.5 mg/kg b.wt.) but was lesser than at high dosage. Cisplatin is known to boost the cytotoxic T-lymphocyte mediated antitumor immunity [7], [8]. Park et al [37] also showed that cisplatin treatment increased the levels of IFN-γ and IL-2. IL-10 has been suggested to play a role in counterbalancing the exacerbated polarized response that may develop following cure [38]. The studies of Lehman et al [39] confirmed that in visceral leishmaniasis a Th1 dominated immune response is protective against *L. donovani* parasites and furthermore, the capacity to produce IFN-γ rather than the presence of IL-4 determines the efficacy of the immune response in susceptible miceHigh levels of IL-4 and IL-10 in control animals supported a view that marked up-regulation of these two cytokines is accompanied by susceptibility, disease progression and depressed Th1 type of cell mediated immunity with decreased production of IFN-γ and IL-12 [40]. Our results confirmed these reports as progressive *L. donovani* infection promote the production of IL-4 and IL-10 and at the same time, suppressed the production of Th1 cytokines such as IFN-γ and IL-2. Similarly in humans, during active VL, the immune response was predominantly of Th2 type, with the absence of IFN-γ in *Leishmania* antigen activated PBMC culture supernatants [41]. So, treatment of mice with cisplatin significantly brought down the IL-4 and IL-10 levels and enhanced the IFN-γ and IL-2 levels after therapy which points towards the shifting of Th2 immune response to Th1 type of immune response, depicting its protective role.

While toxicities induced by cisplatin include ototoxicity, gastrotoxicity, myelosuppression, and allergic reactions [42], [43] the main dose-limiting side effect of cisplatin is nephrotoxicity [44], [45]. Nephrotoxicity increases with the dose and frequency of administration and cumulative dose of cisplatin [44]. As cisplatin causes nephrotoxicity, it has been suggested that the toxic effects of cisplatin may be related to free radical induced damage which can be reduced by the supplementation of antioxidants [46] leading to the possibility that cisplatin may be used in combination with antioxidants which might suppress the drug-induced toxic effects.

To assess the drug induced side effects, various haematological and biochemical studies were carried out. Regarding the hematological parameters, anemia is a frequent manifestation of visceral leishmaniasis that appear with the disease after an incubation period ranging from one month to several years [47] which is in accordance with our study where anemia was found in mice infected with *L. donovani*. Cisplatin treatment at both dosages brought about a significant reduction in the white blood cells and anemia. This is in accordance with an earlier study by Khynriam and Prasad [21] where cisplatin treatment of tumor bearing mice caused a decrease in total leucocytes and severe anemia when given repeatedly Our findings were also in accordance with the studies of Nair et al [48] where body weight, hemoglobin levels and leucocyte counts were decreased after cisplatin injection in mice. In a previous study, a reduction in ototoxicity, renal toxicity, and hematologic toxicity in animals treated with cisplatin plus supplementation with high doses of antioxidants [46] has been reported which is further correlated with our findings where antioxidant supplementation showed no discrepancy in the hematological parameters.

Liver enzymes are the earliest to show a rise even before the appearance of clinical signs (hepatic damage, pulmonary tuberculosis) and their monitoring at various intervals is thus an index of the extent of damage to the liver [49]. It has already been observed that all the medications used to treat visceral leishmaniasis may be associated with significant increase in levels of liver enzymes during treatment which may be due to the killing of the parasites in liver, rather than to direct medication induced hepatotoxic effects [50] and thus hepatocyte damage is considered as a non-desirable side effect [51]. Infected animals treated with cisplatin at the dose of 5 mg/kg b.wt. and 2.5 mg/kg b.wt. showed increase in SGOT, SGPT and LDH levels depicting hepatocellular damage. Hepatotoxicty is a rare side effect of cisplatin. However, it is known that cisplatin is significantly taken up in human liver [52]. Some reports suggest that cisplatin-induced hepatotoxicity may be dose-related [52]. High doses of cisplatin have been found to produce hepatotoxicity, with apoptosis as the major lesion, and metallothionein protects against cisplatin-induced liver injury [53]. Our study has shown pronounced increase in liver enzymes in mice treated with high dosage as compared to those treated with low dosage. The increase in hepatic enzymes is more pronounced in infected and treated animals which may be because the parasite causes structural and functional derangement of liver. Zicca et al [52] showed that the high dose of cisplatin (7.5 mg/kg) administered to rats caused an evident liver damage characterized by significant increase of glutamic oxaloacetic transaminase and γ–glutamyl transpeptidase plasma activities [52]. Furthermore, Lactate dehydrogenase (LDH) enzyme is considered to be a specific marker for tissue damage [54]. LDH is a key enzyme in energy metabolism located in the cell cytoplasm and alkaline phosphatase is a phosphohydrolase enzyme attached to the cell wall by glycosyl phosphatidyl inositol anchors. Activities of these enzymes in urine are physiologically very low. Therefore, any increase in their activities suggests proximal tubular cell damage [55]. Cisplatin treatment at both the dosages brought about increase in LDH level which is related to the studies of Sudhakar et al. [56] where cisplatin treatment significantly increased the enzyme activities of SGOT, SGPT and LDH levels. It has been established that lipid peroxidation might participate in the hepatotoxicity in cisplatin-treated animals despite activation of antioxidant enzymes [57]. It has been suggested that antioxidant enzymes represent the protective response against cisplatin toxicity in the livers of tumor-bearing animals [53]. Many antioxidants have been studied to protect tissue from cisplatin's side effects. In the present study, administration of antioxidants reduced the side effects causing the hepatocellular damage by cisplatin administration. This could be substantiated with the observation of Molander et al. [58], who have also reported that the serum levels of the transaminases return to normal as the liver parenchyma heals and the liver cells regenerates. Silibinin is the most biologically active component with regard to antioxidant and hepatoprotective properties [59]. Pretreating rats and mice with silymarin before exposure to chemical hepatotoxins, such as carbon tetrachloride, thallium, acetaminophen and halothane, significantly reduced lipid peroxidation and hepatotoxicity [60] which is consistent with our study where silibinin administration showed protective response against hepatic damage. Recent work by Seo and Lee [61] provides evidence that low vitamin C intake as ascorbic acid acts primarily as an antioxidant and prevents hepatotoxicity. Both the findings are correlated to our study where supplementation of α-tocopherol and ascorbic acid showed the antioxidant effect and reduced the hepatotoxicity which might be caused by the killing of the parasite or by the administration of cisplatin. SSG treatment brought about a transient increase in SGOT and SGPT which returned to their normal levels within 15 to 30 p.t.d., thereby indicating that drug treatment may cause reversal of damage caused by the parasite and shows recovery of the damaged liver. It may be possible that liver finds it difficult to deal with the load of the drug and parasite but after increase in post treatment days, it may be capable of recovery. Our results clearly support the findings of Crofton and Andrews [62] that liver enzymes improved as patients progressed under chemotherapy.

Cisplatin has been shown to cause nephrotoxicity in patients [63] as well as in a variety of animal species [64]. On studying the renal parameters, increase in levels of serum urea, BUN, uric acid, creatinine and decrease in electrolytes like Mg, Na etc. was observed in the cisplatin (both doses) treated animals. The increase in renal parameters after cisplatin treatment points towards the nephrotoxic effect of the drug. The findings correlate with earlier studies of some workers where a marked increase in blood urea nitrogen and creatinine in serum on treatment with cisplatin at different doses has been reported [65]. The increase of serum creatinine and urea levels was 7 and 5.7-fold, respectively after treatment with cisplatin at a dosage of 16 mg/kg b.wt. [66]. Kaur et al [10] also reported an increase in the renal parameters of mice on cisplatin administration. In our study, loss of electrolytes has been observed, resulting in hyponatremia, hypomagnesemia, hypocalcemia, hypokalemia and hypophosphatemia when infected mice were treated with cisplatin. This decrease was more transient with high dose of cisplatin (5 mg/kg b.wt.). Cisplatin (CDDP) is a well-known chemotherapeutic agent that is associated with hyponatremia. Cisplatin regimens can lead to a more or less pronounced hyponatremia in 4 to 10% of cases due to salt wasting with hypomagnesemia and normokalemia [67]. This is in accordance with our study where cisplatin administration caused hyponatremia in *Leismania donovani* infected mice. However, in relevance to our study moderate hyponatremia (131 mmol/l) without any other biological or clinical disturbances was noticed on day 6 of cisplatin administration in the studies by el Weshi et al. [67].

Hypomagnesemia is a well-known side-effect in patients undergoing chemotherapy with cisplatin containing regimens and very little is actually known about the clinical importance of hypomagnesemia as induced by cisplatin. When cisplatin induces renal injury, declining values of serum magnesium seem to be one of the earliest signs and can be found in the presence of otherwise normal tubular function [68]. Ariceta et al. [69] found, that the minimal cumulative dose required to induce hypomagnesemia was 300 mg/m^2^ of cisplatin. Buckley et al. [70] followed 50 patients receiving cisplatin in doses of 50 mg/m^2^ at four weeks intervals. They found that the incidence of hypomagnesemia increased during treatment from 41% after one course of chemotherapy to 100% in patients receiving six courses of chemotherapy [71] Our results are in accordance to a previous study where administration of cisplatin to dogs resulted in an increase in potassium clearance [72] which leads to hypokalemia. This might result from the proximal tubular injury by cisplatin leading to an increased delivery of sodium, potassium and water to the distal nephron, which creates a sodium-load dependent potassium secretion. Hypocalcemia occurs frequently among patients receiving cisplatin and the actual frequency is probably dependent on the administered dose. Correction of magnesium blood levels usually should improve the hypocalcemia [73]. Although, mild hypocalcemia is reported in high dose cisplatin treatment, severe hypocalcemia has not been reported in low dose cisplatin treatment [74] as in our previous study where administration of cisplatin at low dosage caused no change in calcium levels [10].

Cisplatin induced suppression of renal antioxidant enzyme activity in previous studies [75], [76] suggests that the diminution of these renal antioxidant systems caused by the drug can be prevented by the supplementation with antioxidants. The balance between oxidant and antioxidant system seemed to be disturbed in our study due to cisplatin administration, and to obviate impairment of this balance supplementation of antioxidants prior to administration of cisplatin is required. Cisplatin induced suppression of renal antioxidant enzyme activities has also supported by Ajith et al [75] and Cetin et al [76]. The present study suggested that administration of antioxidants (vitamin C, vitamin E and Silibinin) before cisplatin injection causes the reversal of renal damage. A higher dose of cisplatin was selected in the study to explore the protective effect of the antioxidants when cisplatin caused maximum damage to the kidneys. We found that vitamin C, vitamin E and flavonoid (silibinin) significantly sheltered the cisplatin-induced nephrotoxicity by impairing the antioxidant system. In the study by Weijl et al [77], cancer patients received cisplatin-based chemotherapy, in which half the patients were given a dietary supplement that consisted of vitamin C, vitamin E and selenium. These patients showed recovery with respect to the severity of the nephrotoxicity induced by cisplatin. It has also been shown that both vitamins E and C decreased lipid peroxidation and augmented the activity of antioxidant enzymes in the kidneys of diabetic rats [78]. In another study, lipid peroxide levels were reduced and levels of antioxidant enzymes and thiol compounds were increased following administration of *α*-tocopherol and ascorbic acid in lead-induced oxidative stress [79] It has been reported that administration of cisplatin at the dosage of 6 mg/kg b.wt., intraperitoneally, at an interval of 120 hours, resulted in a significant increase in the concentration of blood urea nitrogen and creatinine. However, when vitamin E and cysteine were administered along with the cisplatin, the results were partially reversed [65], which supports our findings where normal range of blood urea nitrogen, urea and creatinine levels after the administration of cisplatin (5 mg/kg b.wt. and 2.5 mg/kg b.wt.) along with different antioxidants were reported. Our results were also in accordance with the studies of Ajith et al [80] and Appenroth et al [17] where significant reduction in various liver and kidney function tests was reported when both vitamin C and vitamin E were administered together. Silibinin possesses anti-oxidant and membrane-stabilizing properties that have already been elucidated in hepatocytes challenged with a variety of radical-generating drugs [81]. Silibinin partly or totally ameliorated cisplatin induced alterations in parameters associated with proximal tubular function. Administration of cisplatin caused a decline in kidney function within a day following treatment but administration of silibinin caused the reversal of kidney function tests within normal range. Our results were in accordance with the studies of Gaedeke et al [15] where similar results were evaluated. Hypomagnesemia observed by Mavichak et al. [82] was also reported from our study after cisplatin treatment but not after treatment with cisplatin in combination with silibinin signifying its strong antioxidant potential. However, silymarin caused a marked decrease in potassium excretion, suggesting that this constituent is a potassium-sparing diuretic [83]. The above findings correlates with our study where silibinin in combination with vitamins and even alone ameliorated the cisplatin induced alterations in the kidney function tests. Concomitant treatment of antioxidants rendered protection from oxidants attack. All the enzymatic levels which were reported to be higher in the infected controls and the infected plus cisplatin treated animals, ended up being normal in the animals treated with cisplatin and vitamin C, vitamin E and silibinin combination.

Hence, we establish that higher dosage of cisplatin is effective in diminution of parasite burden in visceral leishmaniasis. However, it is recommended that higher dose should be used in combination with antioxidants which help in suppression of drug-induced toxic effects. The results presented in the study are promising in context of reduction in parasite burden, enhancement of immune responses and reduction in the toxic effects. The encouraging results could lead to studies with combination of immunomodulators/herbal extracts and in other animal models for further development of cisplatin as an antileishmanial therapy. To examine the immunomodulatory effect of cisplatin, further studies can be carried in immunosuppressed models.
